# Fetuin exhibits a strong affinity for plutonium and may facilitate its accumulation in the skeleton

**DOI:** 10.1038/s41598-019-53770-6

**Published:** 2019-11-26

**Authors:** Claude Vidaud, Laurent Miccoli, Florian Brulfert, Jean Aupiais

**Affiliations:** 1 CEA, DRF, BIAM-Marcoule, F-30207 Bagnols sur Cèze, France; 2 Laboratoire de RadioToxicologie, CEA, Université de Paris-Saclay, F-91297 Arpajon, France; 3 CEA, DAM, DIF, F-91297 Arpajon, France

**Keywords:** Blood proteins, Nuclear chemistry

## Abstract

After entering the blood, plutonium accumulates mainly in the liver and the bones. The mechanisms leading to its accumulation in bone are, however, completely unknown. We already know that another uptake pathway not involving the transferrin-mediated pathways is suspected to intervene in the case of the liver. Fetuin, a protein playing an important role in bone metabolism, is proposed as a potential transporter of Pu from serum to bone. For the first time, the binding constants of these two proteins (transferrin and fetuin) with tetravalent plutonium at physiological pH (pH 7.0) were determined by using capillary electrophoresis (CE) coupled with inductively coupled plasma mass spectrometry (ICP-MS). Their very close values (log_10_ K_PuTf_ = 26.44 ± 0.28 and log_10_ K_PuFet_ = 26.20 ± 0.24, respectively) suggest that transferrin and fetuin could compete to chelate plutonium, either in the blood or directly at bone surfaces in the case of Pu deposits. We performed competition reaction studies demonstrating that the relative distribution of Pu-protein complexes is fully explained by thermodynamics. Furthermore, considering the average concentrations of transferrin and fetuin in the blood, our calculation is consistent with the bio-distribution of Pu observed in humans.

## Introduction

The actinides, also known as the 5 *f* transition elements, are potent toxicants on both the chemical and radiological levels. As of the early 1940s, the extension of their use for civilian or military purposes led health authorities to focus the research dedicated to actinide biology on the protection of workers and of public health^1^. The description of the behaviour of actinides in biological systems, the precise determination of their bio-distributions in organs and tissues, and their biokinetics in the human body in relationship with their mode of exposure (i.e., inhalation, ingestion, or wounds) have been and remain crucial in understanding the mechanisms that explain their chemical toxicity and the resulting radiological damage. These studies are also essential for designing efficient therapeutic protocols aimed at antagonising or at least limiting their deleterious effects^2,3^. Nevertheless, for all of these particular metals, the molecular events leading to their transport, accumulation, and excretion are rarely, if ever, described.

The above situation applies to plutonium, a synthetic element produced in nuclear reactions. Plutonium is highly redox-active and exists under four oxidation states (III, IV, V, and VI) in environmental conditions. For *in vivo* use, however, the IV oxidation state is preferred^1^, and although Pu^IV^ is very sensitive to hydrolysis and forms colloidal species in aqueous solutions at physiological pH, it is relatively stable when present in cells^4^. This stability facilitates the binding of the metal to biological macromolecules such as proteins, thereby preventing or limiting the process of hydrolysis. Pu^IV^ presents very low clearance and is strongly retained in the human body. Bio-distribution studies indicate that the skeleton and the contents of the liver account for more than 80% of the injected Pu^IV^, with a partitioning in favour of the liver^5,6^. That said, human epidemiological data are scarce, most of the knowledge pertaining to the toxicity of plutonium having been gleaned from experiments performed on animal models. Furthermore, it remains difficult to precisely quantify Pu in human bone: individual variability (age, health status, etc.), the small size of bone samples and their heterogeneous origin (sternum, femur, ribs, scapula, etc.), and the lag time between autopsy and post-mortem sampling are all parameters that require extrapolation and that contribute to the overall heterogeneity of the results.

Pu^IV^ belongs to the group of “hard” cations and prefers hard electron donors such as oxygen. Its charge-to-radius ratio (4.3) is very close to that of Fe^III^ (4.6)^6^. The transport and accumulation properties of Pu^IV^ are also very similar to those of Fe^III^. It associates *in vivo* with the proteins involved in iron metabolism such as serum transferrin (Tf) and ferritin^7,8^. Transferrin is a glycoprotein of around 78 kDa with an isoelectric point of 6.3, and its average concentration in serum is 2.5 mg/mL (~30 µM). It is responsible for transporting iron from the blood towards the various organs, in particular the liver. This protein has two lobes, the C- and the N-lobe, in which two Fe^III^ can be strongly bound (log_10_ K_1_ = 21.4 and log_10_ K_2_ = 20.3, respectively)^9^. The carbonate ion serves as a synergistic anion, ensuring the closure of the lobes and strengthening the binding of the metal to Tf. Only the di-ferric form of the protein is properly conformed to be selectively internalised into the cells by receptor-mediated endocytosis. The apo form and the mono-ferric form of the protein represent around 70% of the protein in normal serum^10^, and several transition metals or even lanthanides have been shown to bind to either one or both of the C- and N-lobes, their binding constants being affected by the concentration of the synergistic anion bicarbonate^9^. In particular, Tf can bind Pu^IV^ into its two lobes^11^ with a reported conditional constant p*K*_*d*_ of 21.25 ± 0.75^12^. Recently, Sauge-Merle *et al*.^13^ used capillary electrophoresis coupled with an inductively coupled plasma mass spectrometer (CE-ICPMS) to investigate the binding constant of human Tf for Pu^IV^, and found log_10_ K*_1_ = 25.0 at pH 6, 0.1 M NaCl, and 25 °C. Therefore, even though another uptake pathway not involving the Tf receptor-mediated system has been suggested as a source of intracellular Pu, this system can partially explain the accumulation of Pu^IV^ in the liver^14–16^.

Recently, an elegant set of studies by Jensen and colleagues found that mammalian cells could acquire Pu^IV^ through the common Fe uptake pathway of receptor-mediated endocytosis of metallo-transferrins. In this study, Pu needed Fe in order to be taken to the cells, because only the Tf complex containing one Pu on the C-lobe and one Fe on the N-lobe was recognised. All other forms of plutonium-Tf did not adopt the correct conformation for receptor recognition and uptake^17,18^.

But Tf by itself cannot explain why Pu^IV^ accumulates in the bones. Bone is a specialised, highly vascularised connective tissue controlled by a finely-regulated cellular system ensuring its growth and homeostasis. It is composed of a protein framework, containing mainly collagen but also non-collagenous proteins, permeated with a mineral matrix of hydroxyapatite and salts such as magnesium. These non-collagenous proteins are either expressed *in situ* by the bone cellular system or released from the bloodstream. Among the non-collagenous proteins in serum, fetuin-A (Fet, also named α2-HS-glycoprotein), represents more than 25% (in mass)^19^. Fetuin is by far the main protein, because it is selectively enriched from serum and is four times more concentrated than albumin in the calcified matrix^20^. Fetuin is a liver-derived plasma protein. It is the most abundant globular plasma protein in fetuses and young children, its serum level then declining over time and finally stabilising at around ~15 µM in adults^21^. Its best-known function pertains to mineralisation biology. It “buffers” mineral ion supersaturation by binding small clusters of calcium and phosphate that could be abnormally formed in the circulation^22–24^. Thus it acts as an important circulating inhibitor of ectopic calcifications^25^ by carrying these calciprotein particles (CPP) to the organic matrix of the bone, where its life cycle ends around 1.5 days after its release into the bloodstream^20^.

Human Fet has a 48 kDa theoretical molecular mass, but is highly glycosylated, leading to apparent molecular masses of up to around 56 kDa^26^. It is composed of two conserved cystatin domains, D1 and D2, and an unstructured D3 domain^19^. The D1 domain contains acidic clusters that can be prone to hard Lewis binding. Recently, we demonstrated that Fet is able to convey more than 80% of the hard cation uranyl that is bound to the serum proteins, making it the major carrier of uranyl in blood^27^. Fetuin can bind three uranyl ions with an apparent overall *K*_D_ of around 30 nM^28^. The strong binding constant (log_10_ K = 11.4) of the first site was determined by CE-ICP-MS analysis^29^.

We therefore hypothesised that if the binding constant of Fet was high enough to allow its successful competition with Tf, Fet could, by binding to Pu^IV^, participate in the transport of Pu to the bones. A coupling between capillary electrophoresis and inductively coupled plasma mass spectrometry (CE-ICP-MS) was performed to monitor metal binding on the proteins. This coupling is advantageous because it requires only low quantities of both protein and metal, while providing very sensitive detection of Pu by ICP-MS (with a detection limit as low as 1.10^−12^ M). In accordance with previously published results^13^, a coated capillary was used to prevent the capillary from becoming clogged with proteins, and Pu speciation was controlled through the use of the nitrilotriacetate (NTA) anion to prevent Pu hydrolysis. First we calculated the binding constants of Pu-Tf and Pu-Fet at pH 7.0 using competitions with Pu(NTA)_2_; then we established a competition for Pu^IV^ between Fet and Tf by increasing the Fet concentration while maintaining constant that of the Pu-Tf complex. Taking into account their respective concentrations in serum and their binding constants, a distribution of Pu between the two proteins can be calculated. The results obtained are discussed with a focus on the partitioning of Pu between the bones and the liver.

## Results and Discussion

### Electropherograms of Pu-transferrin and Pu-fetuin

**Figure 1:**
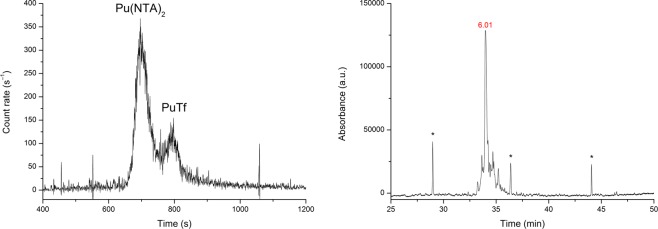
left – Electropherogram of Pu/NTA/Tf at pH 6.0. Sample: [Pu] = 0.1 nM, [NTA] = 1 µM, [Tf] = 47 nM, NaCl/MES buffer, I = 0.1 M. Separation conditions: 25 °C, V = +5 kV, N-CHO^TM^ capillary from Beckman Coulter, L = 65 cm, internal diameter 50 µm; right – CIEF electropherogram of apoTf (no plutonium present); *pI marker.

**Figure 2:**
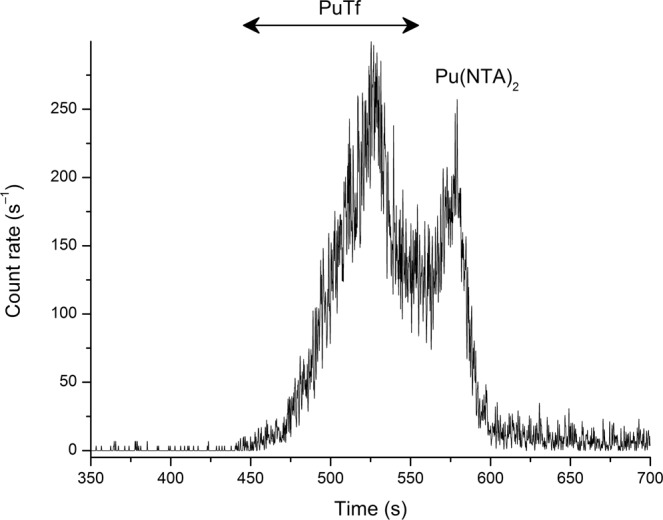
Electropherogram of Pu/NTA/Tf at pH 7.0. Sample: [Pu] = 1 nM, [NTA] = 2 µM, [Tf] = 10 µM, NaCl/TRIS buffer, I = 0.1 M. Separation conditions: 25 °C, V = +7 kV, N-CHO^TM^ capillary from Beckman Coulter, L = 65 cm, internal diameter 50 µm.

The PuTf electropherograms obtained at pH 7.0 are quite different from those obtained at pH 6.0 in a previous study^13^. At pH 6.0 (see Fig. 1, left), the PuTf species is located at the right side of the anionic species ${\rm{Pu}}{({\rm{NTA}})}_{2}^{2-}$ and presents a typical Gaussian-shaped profile with no fine structure. At pH 6, Tf is neutral because its isoelectric point is near pH 6.3. Capillary isoelectric focusing (cIEF) was carried out, showing an experimental isoelectric point at pI = 6.0 (Fig. 1, right). At pH 7.0, Tf is therefore slightly negatively charged and is localised at the left side of Pu(NTA)_2_ (see Fig. 2). In addition, the charged protein undergoes an electrophoretic effect, leading to a rough unfolding of its fine structure, as shown in Fig. 2. The migration times for both species are also shortened, because the magnitude of electroosmotic flow increases with pH.

**Figure 3:**
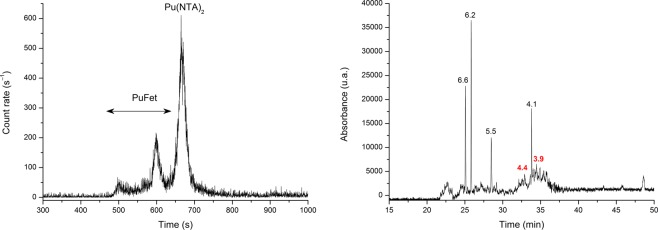
left – Electropherogram of Pu/NTA/Fet at pH 7.0. Sample: [Pu] = 1 nM, [NTA] = 2 µM, [Fet] = 8 µM, NaCl/TRIS buffer, I = 0.1 M. Separation conditions: 25 °C, V = +7 kV, N-CHO^TM^ capillary from Beckman Coulter, L = 62 cm, internal diameter 50 µm; right – cIEF electropherogram of Fet without plutonium. The protein presents two distinct peaks at pI = 4.4 and 3.9.

An example of separation for the PuFet system is given in Fig. 3. The existence of two peaks is consistent with the cIEF spectrum, in which Fet shows two distinct pI values (3.9 and 4.4, respectively). Under our separation conditions, i.e., pH 7.0, the Fet is negatively charged.

### Determination of the binding constants

The main difficulty when working with tetravalent plutonium at physiological pH is its extreme sensitivity to hydrolysis^30^. The protection of Pu^IV^ against hydrolysis requires the formation of a stable species at pH 6–7. The nitrilotriacetate anion was used because it presents two major advantages: it has a strong affinity for plutonium (log_10_ β_2_ = 37.90 for the reaction ${{\rm{Pu}}}^{4+}+2{{\rm{NTA}}}^{3-}\rightleftharpoons {\rm{Pu}}{({\rm{NTA}})}_{2}^{2-}$)^31^ and for Tf it acts as a synergistic anion^32^.

We applied the methodology previously published^13^. The Pu and NTA concentrations were fixed, and the Tf concentrations were varied from 1 µM to 31 µM. The NTA concentration was adjusted to 2 µM (instead of 1 µM in our previous paper) because the experimental pH was increased to 7.0.

According to data previously published^31^, ${\rm{Pu}}{({\rm{NTA}})}_{2}^{2-}$ is the main species in solution (100%) at pH = 7.0 and C_NTA_ = 2 µM. Therefore, we considered only the equilibrium below (with charge omitted):

1 $$Pu{(NTA)}_{2}+Prot\rightleftharpoons PuProt+2NTA,$$

with Prot = Tf (transferrin) or Fet (fetuin-A).

2 $${K}^{\ast },\,the\,conditional\,constant,\,is\,defined\,as{K}^{\ast }=\frac{[PuProt]{[NTA]}^{2}}{[Prot][Pu{(NTA)}_{2}]}$$

At the inflexion point of the sigmoidal function, the [Pu(NTA)_2_] and [PuProt] concentrations are equal, which simplifies Eq. (2) to:

3 $${K}^{\ast }=\frac{{[NTA]}^{2}}{[Prot]}=\frac{{({C}_{NTA}/a)}^{2}}{{C}_{Prot}},$$

with C_NTA_ and C_Prot_ the total concentrations of ligand, and the coefficient α defined as:

4 $$\begin{array}{rcl}\alpha  & = & \frac{{C}_{NTA}}{[NT{A}^{3-}]}=1+{10}^{pk{a}_{4}}[{H}^{+}]+{10}^{pk{a}_{4}+pk{a}_{3}}{[{H}^{+}]}^{2}\\  &  & +{10}^{pk{a}_{4}+pk{a}_{3}+pk{a}_{2}}{[{H}^{+}]}^{3}+{10}^{pk{a}_{4}+pk{a}_{3}+pk{a}_{2}+pk{a}_{1}}{[{H}^{+}]}^{4}\end{array}$$

where pka_i_ stands for the four stepwise dissociation constants of nitrilotriacetic acid. Under our experimental conditions (pH = 7.0), *log*_10_α is equal to 2.68 ± 0.04.

From Eq. (3) and the constant of formation of Pu(NTA)_2_, the conditional constant relative to the formation of PuTf can be deduced:

5 $$lo{g}_{10}{K}_{PuProt}^{\ast }=lo{g}_{10}{K}^{\ast }+lo{g}_{10}{K}_{Pu{(NTA)}_{2}}$$

### Pu-transferrin system

In the case of Tf, the comparison with other data must take into account the concentration of bicarbonate anions. This correction is applied by means of the bicarbonate-independent binding constant (${{\rm{K}}}_{1}^{\ast }$)^33^:

6 $$lo{g}_{10}{K}_{1}^{\ast }=lo{g}_{10}{K}_{PuTf}^{\ast }+lo{g}_{10}{\alpha }_{c},$$

where *α*_*c*_ is the fractional saturation of the human apo-Tf binding sites with bicarbonate according to the equation:

7 $${\alpha }_{c}={K}_{c}\frac{[HC{O}_{3}^{-}]}{1+{K}_{c}[HC{O}_{3}^{-}]},$$

with *K*_*c*_ the binding constant of bicarbonate with Tf.

At equilibrium with the atmosphere, the concentration of bicarbonate can be easily determined. As a result, $lo{g}_{10}{\alpha }_{c}$ = −2.27. The results of the calculation are given in Table 1.

**Table 1:** Experimental conditional binding constants K* at pH 7.0, I = 0.1 M, 25 °C.

Equilibrium	*log*_10_ K*
$Pu+Fet\rightleftharpoons PuFet$	26.20 ± 0.24
$Pu+Tf\rightleftharpoons P{u}_{C}Tf$ $lo{g}_{10}{K}_{1}^{\ast }=lo{g}_{10}{K}_{PuTf}^{\ast }+lo{g}_{10}{\alpha }_{c}$	26.44 ± 0.28 $log{K}_{1}^{\ast }$ = 24.17 ± 0.30

### Pu-fetuin system

**Figure 4:**
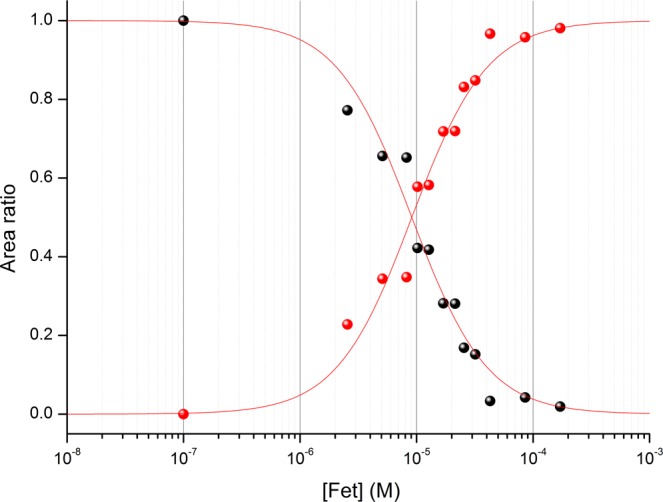
Effect of increased concentration of Fet on the Pu(NTA)_2_ complex. The relative area of Pu(NTA)_2_ (black symbols) is reported as a function of PuFet (red symbols). The equivalence point is found for [Fet] = (9.1 ± 1.3) µM. Separation conditions: 25 °C, V = +7 kV, N-CHO^TM^ capillary from Beckman Coulter, L = 62 cm, internal diameter 50 µm, C_NTA_ = 2 µM, pH 7.0, NaCl/TRIS buffer, I = 0.1 M, [Pu^IV^] = 1 nM.

Based on the electropherograms obtained at pH 7.0 (see Fig. 3), the relative areas between the species Pu(NTA)_2_ and PuFet were calculated for increasing concentrations of Fet (see Fig. 4). The intersection point was found to be [Fet] = (9.1 ± 1.3) µM, corresponding to a conditional binding constant of log_10_ K* = 26.20 ± 0.24 (see Table 1). This value is almost identical to the one calculated for the formation of PuTf (log_10_ K* = 26.44 ± 0.28).

**Figure 5:**
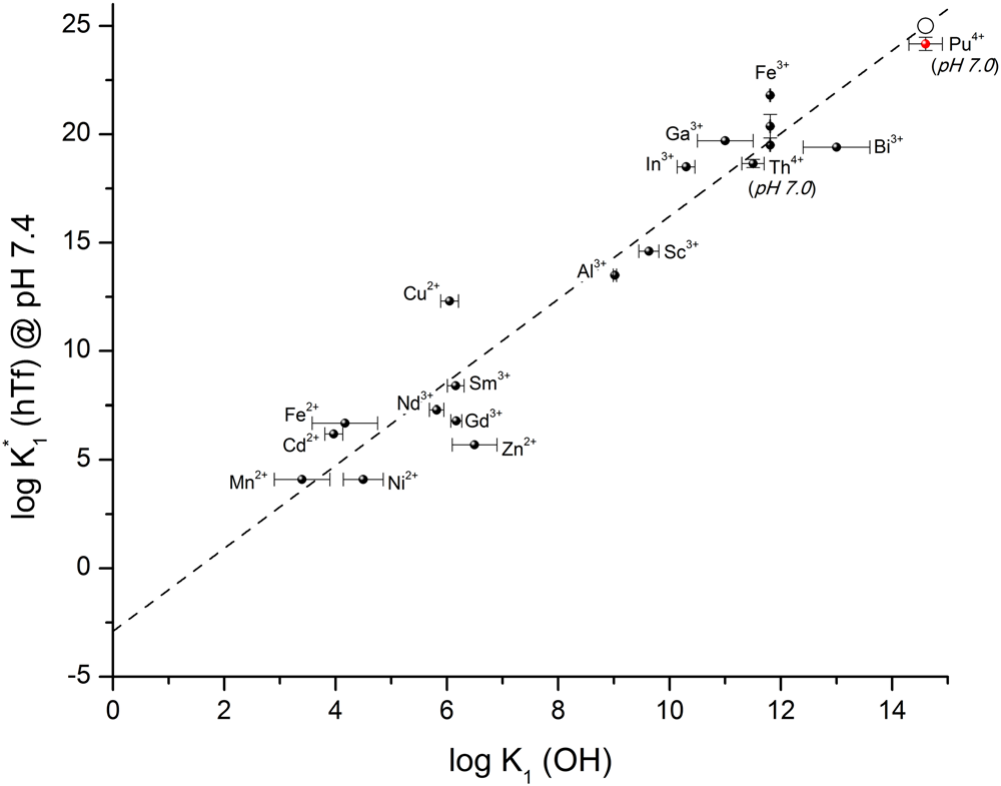
Variation of the conditional constant ${K}_{1}^{\ast }$ as a function of the first hydrolysis of metal^13,44^. At pH 7.4 (○), the binding constant is expected to be log_10_ ${{\boldsymbol{K}}}_{1}^{\ast }$ = 25.0. At pH 7.0, the value 24.17 ± 0.30 was found (red point).

In a previous paper, the conditional binding constant between tetravalent plutonium and Tf was determined at pH 6.0. This choice of pH was initially justified by the difficulty of protecting the tetravalent cation against hydrolysis^13^. Because the binding constant is expected to increase with pH as published elsewhere^34^, a new determination was performed as close as possible to the physiological pH. Based on the binding constants recently re-evaluated in the literature for the interaction between plutonium and the nitrilotriacetate anion^31^, it appeared that experiments at physiological pH (7.4) could not be performed, because pH 7.0 was the upper value satisfying the criterion relative to protection against hydrolysis. Above this pH value, hydrolysis could not be avoided. Nevertheless, we considered this pH of 7.0 to be close enough to the physiological pH to be representative in terms of chemical behaviour. Figure 5 shows that the experimental value is close to that expected based on the correlation between the first hydrolysis constant and ${K}_{1}^{\ast }$^9^.

### Competition between transferrin and fetuin at pH 7.0

According to Table 1, Fet and Tf present a similar affinity for plutonium. From these very interesting results, we can hypothesise that these two proteins may be competing for Pu in serum. In order to confirm this hypothesis, competition reactions were carried out with various protein concentration ratios (see Table 2). We can assume that if thermodynamics drives the competition between these proteins, then the relative distribution of plutonium between them will obey the law of mass action.

**Table 2:** Variation of the $\frac{[{\boldsymbol{PuTf}}]}{[{\boldsymbol{PuFet}}]}$ ratio as a function of the concentrations of fetuin and transferrin.

Set	[Transferrin] (in µM)	[Fetuin] (in µM)	$\frac{[{\boldsymbol{PuTf}}]}{[{\boldsymbol{PuFet}}]}$
1	3	8	0.93
	5	8	0.95
	0.3	8	0.18
2	0.086	0.86	0.14
	0.086	0.43	0.35
3	10	8	1.44
	1	8	0.41

Conditions: [Pu] = 1 nM, C_NTA_ = 2 µM, pH 7, 25 °C. Three sets of experiments were performed on three different weeks within two months. Each set of experiments is independent with new solutions, reagents, buffers, and capillaries.

Thus,

8 $$\frac{[PuTf]}{[PuFet]}=\frac{{K}_{PuTf}^{\ast }[Pu][Tf]}{{K}_{PuFet}^{\ast }[Pu][Fet]}=\frac{{10}^{26.44}}{{10}^{26.20}}\times \frac{[Tf]}{[Fet]}$$

9 $$\frac{[PuTf]}{[PuFet]}=1.738\times \frac{[Tf]}{[Fet]}.$$

At equilibrium, the $\frac{[PuTf]}{[PuFet]}$ ratio increases linearly with the $\frac{[Tf]}{[Fet]}$ ratio by a constant factor of 1.738.

**Figure 6:**
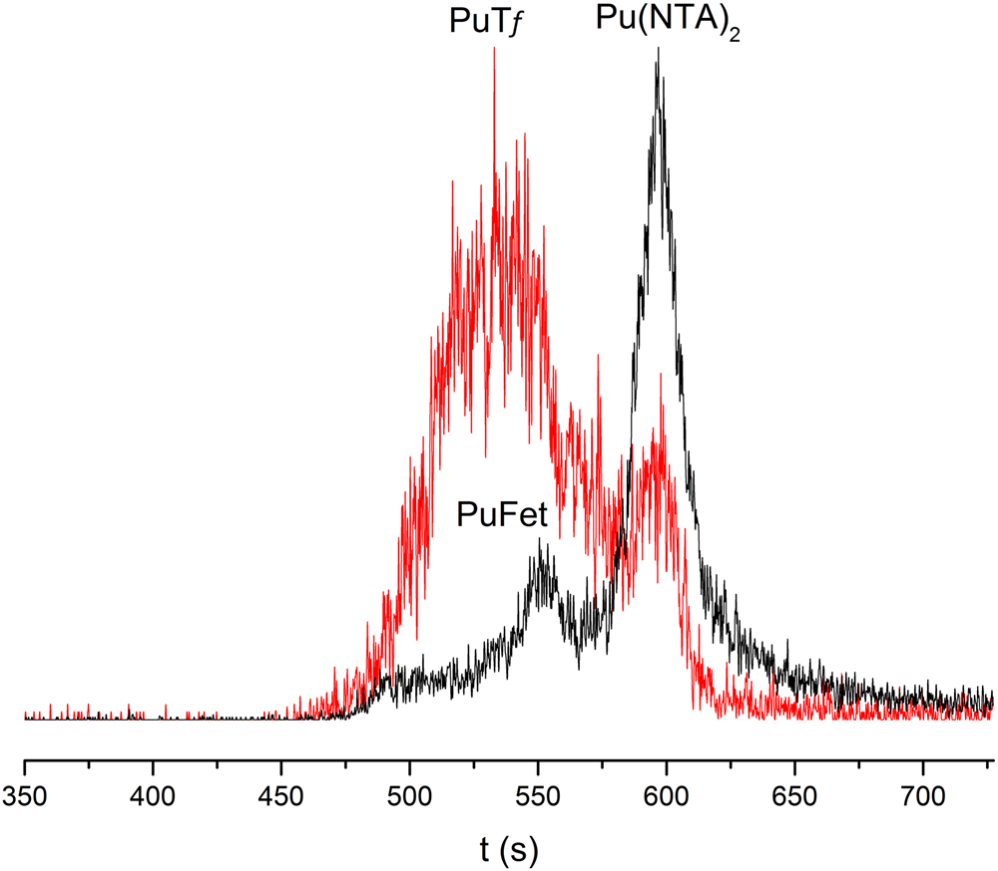
Electropherograms of a Pu/NTA/Fet/Tf mixture at pH 7.0. Red [Fet] = 8 µM + [Tf] = 10 µM, black [Fet] = 8 µM (without Tf). Sample: [Pu] = 1 nM, [NTA] = 2 µM, NaCl/TRIS buffer, I = 0.1 M. Separation conditions: 25 °C, V = +7 kV, N-CHO^TM^ capillary from Beckman Coulter, L = 62 cm, internal diameter 50 µm.

In practice, both proteins were first mixed together in the appropriate ratio, and then a Pu aliquot solution stabilised by NTA was added. The final mixture was analysed by CE-ICPMS. The example given in Fig. 6 shows a co-migration of Pu-protein complexes at pH 7.0. In order to determine each contribution, additional experiments, either without Fet or without Tf, were performed within the same set of experiments. All results are gathered in Table 2.

**Figure 7:**
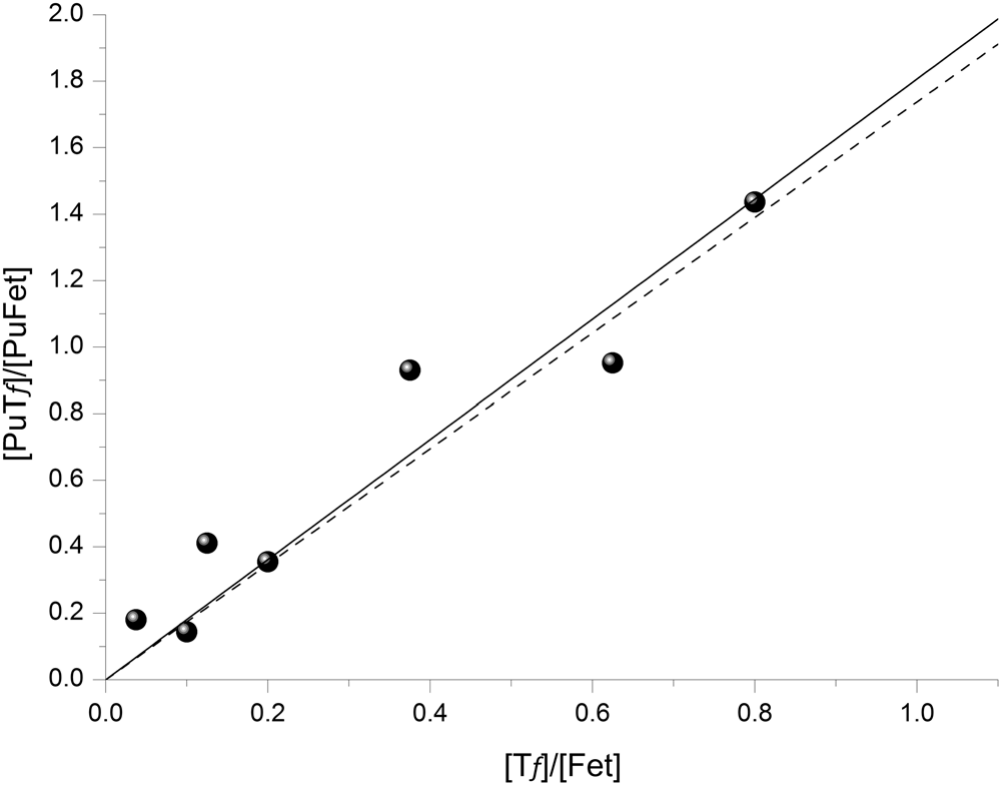
Variation of $\frac{[{\boldsymbol{PuTf}}]}{[{\boldsymbol{PuFet}}]}$ as function of $\frac{[{\boldsymbol{Tf}}]}{[{\boldsymbol{Fet}}]}$; dashed line corresponds to expected slope based on binding constants with log_10_ K_PuTf_ = 26.44 and log_10_ K_PuFet_ = 26.20 (slope = 1.738); straight line corresponds to linear regression based on data slope = 1.81 (•).

The slope calculated from the data in Table 2 (1.81 ± 0.27) is in excellent agreement with the expected slope (see Fig. 7). We can conclude that the chemical equilibria are attained and that the distribution of plutonium between these two proteins is driven only by the binding constants.

One can estimate a theoretical bio-distribution of Pu between the liver and the bones from the relative affinity of Tf and Fet for Pu. Using the average concentrations of Fet (15 µM) and of the unsaturated forms of Tf (70% of 30 µM) in the blood, one can calculate the relative ratio of plutonium bound to these proteins (Eq. 9). It leads to $\frac{[PuTf]}{[PuFet]}$ ∼2.5, i.e., a relative distribution of ∼71% for PuTf and ∼29% for PuFet. But we have to keep in mind that the concentration of these two proteins can also vary in normal serum. For example, Tf concentrations can vary from 25 to 51 µM in a healthy human, which leads to 17.5 to 35.7 µM for the unsaturated forms. These variations can lead to $\frac{[PuTf]}{[PuFet]}$ values from ∼2 to ∼4, i.e., a relative percentage distribution of PuTf and PuFet that varies from 67/33 to 80/20. Furthermore, this theoretical biodistribution is probably displaced toward a higher PuFet content since only one form of unsaturated transferrin over the three ones (one fully unsaturated, 2 different Fe-Tf complexes) can lead to the only one (Fe/Pu)-Tf conformation adapted to be recognized by the transferrin receptor 1.

These results must be compared with the *in vivo* data available from the literature. Few papers deal with Pu distribution in the case of human contamination. Stather and Nenot^35^ summarised the data from the literature, which exhibit variations due to inherent individual variability and sample heterogeneities, as explained in the introduction. Based on about 200 samples, however, the distribution between the liver and the bones was found to be 0.57 and 0.23 pCi.kg^−1^, respectively. Other data were given for the lungs and lymphatic glands but here, for the purposes of the present discussion, we are considering plutonium originating from blood after a contamination. The activities found in the lungs, the liver, and the vertebra did not deviate from a factor of two or three^35^. Thus, based on these data, the relative distribution of Pu in humans is in the range of around 71% for the liver and 29% for bone. In addition, five studies performed in the 1970s using 25 autopsies from strongly contaminated workers showed a relative average distribution of 72% in the liver and 28% in the bones, but with wide variation among individuals (liver 16–95%; bone 5–85%)^35^. In a draft of the update of the ICRP publication “Occupational Intakes of Radionuclides: Part 4,” a 50/50 distribution of Pu between the liver and the bones was described in humans, as in the case of healthy Mayak workers who had inhaled Pu. The considerable disparities observed between the liver and the bones were also due to the wide variety of contaminants in the various registers of contaminated workers. Our relative distribution of PuTf and PuFet determined in this paper is consistent with those reported in the literature.

Thus, the physiological variations of the Tf and Fet concentrations between individuals can, to some extent, also contribute to explaining these heterogeneous Pu distributions. An important point is that Fet concentration in infants and young children can exceed 20 µM, whereas their Tf concentration ranges are similar to those of adults. This fact could also explain the greater Pu accumulation observed in the bones of young organisms^36^.

Since the beginning biochemical studies dedicated to Pu toxicity, Pu-Tf complexes were identified as the main bioavailable species responsible of its accumulation in its target organs. In this study conducted by Stevens *et al*.,^37^ the intravenous injection of Pu^4+^ in citrate buffer in beagles resulted in the binding of plutonium to Tf and albumin, with a vast majority of the actinide binding to Tf. When plutonium citrate was injected intravenously into rats, plutonium in the blood serum was shown to bind to proteins, primarily Tf^38^. In another paper, the mechanism of the incorporation of Pu^4+^ by mammals was believed to involve predominantly Tf^39^. In this last paper, 5 min. after intravenous injection of Pu citrate into the rat, only one protein peak (molecular mass around 69.5 kDa) associated with Pu activity was detected by size-exclusion chromatography; it represented around 85% of the total injected activity. Anion-exchange chromatography revealed that the peak could be attributed to Tf, and affinity column chromatography with antibodies against Tf confirmed the qualitative presence of Tf. One can wonder why fetuin was not identified as a Pu carrier in these experiments. Fetuin was first discovered by Pedersen in 1944^40^. First of all, size-exclusion or anion-exchange chromatography is not resolutive enough to directly isolate one or another of these proteins (albumin, transferrin, or fetuin) from a mixture as complex as the serum. Thus, the presence of Pu in a chromatography peak cannot be attributed to a single protein. Fetuin has a large hydrodynamic radius because it is highly glycosylated, and thus it displays an apparent molecular mass within 50–56 kDa. Its isoelectric point is close to that of albumin. Its concentration is rather low, however, compared to those of albumin and transferrin. All of these biophysical characteristics explain why fetuin was first isolated thanks to multiple separation techniques and chromatographic steps, and why it remains difficult to isolate fetuin from albumin even in capillary electrophoresis^29^. Its main biological role (i.e., bone calcification) has, since the 1960s–1980s, been the subject of numerous works dealing mainly with the biochemical mechanisms leading to osteogenesis, bone growth, and bone homeostasis. This could explain why its role in toxic metal accumulation in bone has not been further investigated. Otherwise, the isolation of pure proteins from serum requires fractionation methods followed by multiple chromatographic steps, and the literature concerning the binding of Pu to serum proteins is now very dated. Proteomic methods using mass spectrometry could help to better identify the proteins co-eluted with Pu in a chromatographic peak. Although the results reported in the present paper were obtained *in vitro*, the identification of the proteins conveying Pu to the skeleton should be reconsidered in the light of our results.

## Conclusions

Due to the high radiotoxicity of plutonium, the only analytical tools that allow the chemical behaviour of plutonium species to be investigated are those that involve the coupling of mass spectrometry with separation techniques. Because separation by capillary electrophoresis is not intrusive (the speciation is preserved), CE-ICP-MS is an ideal tool for studying the interaction of plutonium with proteins. The studies of the interactions of plutonium with Tf and Fet proteins at near-physiological pH were successful. They provided fruitful information suggesting that the driving force between both proteins is only thermodynamic. This observation led us to postulate that the fate of Pu in a body depends mainly on the relative concentrations of these two proteins in the blood. That said, the Tf-mediated system cannot explain by itself the accumulation of Pu in bone. In the circulation, iron is bound to Tf, with most of the Tf-bound iron being utilised for bone marrow erythropoiesis (around two-thirds of the 3–5 g of total iron in the body), and Tf receptors are strongly expressed in erythroblasts. The cells able to store iron are mainly the macrophages of the liver, but also the cells of the bone marrow, the spleen, and the muscles. Finally, the remaining amount of iron is distributed between the hepatocytes, for haemoglobin synthesis, and also in the myoglobin of the muscles^41^. The Tf-Tf receptors are involved in all of these processes. A few reports indicate that iron is necessary for osteoblasts, which are the cells responsible for bone accretion^42^. But in fact, little is known of the role of iron in the processes of bone formation. Some data indicate that the amount of iron in the bones of various mammals ranges from several milligrams to a few hundred milligrams of iron per kilogram dry mass^43^, which is very low compared to the amount present in the erythrocytes or the liver.

To conclude, even if the involvement of the Tf-mediated system cannot be totally excluded for bone cell functions, the accumulation of Pu in bone in such a proportion cannot be attributed to Tf alone. On the contrary, Fet is very highly concentrated in the bone matrix, and its high affinity for Pu could now convincingly explain Pu accumulation in the bones. We propose that Pu is conveyed to the bones not only through the citrate species present in serum, but through Fet as a protein carrier.

## Experimental Section

### Safety precaution

All isotopes of plutonium are highly radioactive alpha-emitting radionuclides that must be handled in a specially**-**designed radiological laboratory and require special caution and radiation protection.

### Reagents

Solutions were prepared with ultrapure water (18.2 MΩ.cm; Milli-Q station, Merck Millipore, Merck KGaA, Darmstadt, Germany). All experiments were performed using a background electrolyte (BGE) containing 75 mM NaCl, 25 mM TRIS, and 2 µM nitrilotriacetic acid (NTA). The pH was adjusted to pH 7.0 using NaOH. The ionic strength was 0.1 M. The solution of ^242^Pu in BGE was prepared from a stock solution in 4 M HNO_3_. An aliquot of 1 mL volume was evaporated to dryness, then 500 µL of 12 M HCl was added, and the solution was once more evaporated to dryness. The addition of HCl followed by evaporation was repeated three additional times. After the last evaporation, the sample was cooled to room temperature, and 100 µL of BGE was added. The final concentration of Pu stock solution was 1.10^−9^ M. The metal-binding ligands, Tf and Fet, were prepared as described below. All chemicals were obtained from commercial manufacturers and used as supplied.

### Protein purifications

Commercial human apoTf (T2252, purity >98%) and bovine Fet (F2379) were purchased from Sigma-Aldrich. ApoTf was extensively dialysed against a mixture of 50 mM TRIS and 150 mM NaCl at pH 7.0 and extemporaneously placed in the working buffer. Bovine Fet was first extensively dialysed against a mixture of 50 mM TRIS and 150 mM NaCl at pH 7.0 and then further purified by size-exclusion chromatography (TSKgel®SW 3000 column, 21.5 × 300 mm, 4 mL min^−1^ flow rate) using the same buffer as the mobile phase. Its purity was checked by electrophoresis (SDS PAGE) and mass spectrometry. The final concentrations of the two proteins (mg per mL) were calculated from their absorbance at 278 nm using their molar extinction coefficient (Ɛ = 80 000 and 19 840 M^−1^ cm^−1^, respectively).

### CE-ICP-MS

A Beckman Coulter P/ACE 800 Plus commercial Capillary Electrophoresis (CE) system equipped with a diode array detector (Beckman Coulter, Fullerton, USA) was used for all of the measurements. The measurements were carried out using two kinds of commercially-coated silica capillaries (N-CHO capillary, from Beckman Coulter), with a 50 𝜇m internal diameter, 62 cm total length, and 10.1 cm optical window. The capillaries were preconditioned by rinsing with BGE before use at 10 psi for 5 min. The CE system was provided with a tailor-made capillary cartridge support designed to allow the connection of an external detector, *i.e*., an X Series^II^ ICP-MS (Thermo). Both pieces of apparatus were coupled by a commercial interface using a parallel path micro-nebuliser (Mira Mist CE, Burgener, Mississauga, Canada) specially designed for capillary electrophoresis. A make-up liquid (2% HNO_3_ and 10% ethyl alcohol, absolute) was introduced by means of a syringe pump (11 Pico Plus, Harvard Apparatus, Holliston, MA) at a nominal flow rate of 7 μL.min^−1^ to improve the signal binding by (i) decreasing the surface tension of the water droplets and the size of the droplets and (ii) providing the nominal flow rate for the nebuliser. Samples were injected at the capillary inlet over a period of 4 s at a constant pressure of 2 psi. Separations were performed at +7 kV, 25 °C, and a constant pressure of 1.2 psi to prevent capillary clogging. The voltage value was chosen with respect to Ohm’s law and to prevent temperature increases exceeding 1 °C during the experiments. It should be pointed out that the temperature never decreased below 25 °C but could rise to 26 °C by Joule heating, for the highest electrolyte conductivity. The buffer was changed every run to prevent the effects of electrolysis. Before the injection of each sample, the following washing sequence was performed: 10 mM NaOH at 20 psi for 1 min, then water at 20 psi for 1 min, and finally buffer electrolyte at 20 psi for 1 min.

### CIEF

A Beckman Coulter P/ACE MDQ Capillary Electrophoresis system (Beckman Coulter, USA) equipped with an L2D2 deuterium lamp (Hamamatsu Photonics, Japan) controlled by System Gold Software (Beckman) was used in this study. The cIEF was performed using a 30 cm eCAP neutral capillary (50 µM ID, Beckman) with a 20 cm separation length. The separations were carried out at 25 °C with an optical detection at 280 nm. Capillary conditioning was performed by rinsing the capillary with acetic acid (chemical mobiliser, Sigma-Aldrich) for 5 min at 50 psi, followed by milliQ water (2 min, 50 psi), and then cIEF gel (Beckman) for 5 min at 50 psi. The sequence was concluded with the submersion of both ends of the capillary in milliQ water.

The anolyte consisted of 200 mM phosphoric acid (Sigma-Aldrich) and the catholyte consisted of 300 mM sodium hydroxide (Sigma-Aldrich). The Tf sample (15 µL of approximately 10 mg.mL^−1^) was mixed with 200 µL of cIEF gel, 15 µL of carrier ampholyte (40% Roti®lyte 4–7, Carl Roth GmbH), 25 µL of a 500 mM arginine solution (cathodic stabiliser, Sigma-Aldrich), and 1.5 µL of the required pI marker solution (pI markers 7, 5.5, and 4.1 from Beckman and pI markers 6.6 and 6.2 from Sigma-Aldrich). The Fet sample (15 µL of approximately 15 mg.mL^−1^) was mixed with 200 µL of cIEF gel, 15 µL of a mixture of Roti®lyte 3–5 (Carl Roth GmbH) and Roti®lyte 4–7 (Carl Roth GmbH), 25 µL of a 500 mM arginine solution, and 1.5 µL of the required pI marker solution.

For the separation procedure, the capillary was filled with the mixture previously described by applying the high-pressure rinse mode (25 psi) for 99 seconds. Then, a voltage of 25 kV was applied for 25 min to focus the ampholytes and samples; the cathode was placed in the NaOH solution (catholyte) and the anode in the phosphoric acid solution (anolyte). Since the sample was introduced into the capillary by filling it entirely with a mixture of ampholytes and samples, the amount of sample injected could easily be controlled by adjusting the concentration of the sample in the mixture. Once the focalisation step was completed, the voltage was increased to 30 kV and the catholyte was replaced by the chemical mobiliser (acetic acid, 350 mM). The disruption of the pH gradient caused by the chemical the 200 µm window of the capillary cartridge.

Between analyses, the capillary was rinsed with a 4.5 M urea solution and milliQ water in order to ensure the cleanliness of the capillary and prevent protein aggregation.

The linearity of the pH gradient was verified before the analysis and checked at least once a day. The linearity of the gradient was determined by plotting the pI of the markers versus their retention time. Typically a R² = 0.995 or higher was obtained, showing the good linearity of the pH gradient. This linearity ensures the accuracy of pI estimation, especially when the cIEF method is used for analysis of samples with unknown pI.

### Pu-protein sample preparation at various concentrations for CE-ICP-MS

A solution of apoTf or Fet was dialysed three times against a mixture of 25 mM TRIS, 75 mM NaCl, pH 7.0 buffer. The PuTf samples were prepared as follows: each vial contained BGE, 2 µM NTA, 0.1 nM ^242^Pu, and Tf concentrations varying from 1 µM to 31 µM in a final volume of 100 µL. The PuFet samples were prepared as follows: each vial contained BGE, 2 µM NTA, 0.1 nM ^242^Pu, and Fet concentrations varying from 0.1 µM to 200 µM in a final volume of 100 µL. Although the equilibria were reached for both proteins within a few minutes, they remained in contact with the metal for 15 minutes before use.

### CE-ICPMS data treatment

The previously published procedure was applied^13^. Origin 7.0 Pro software (OriginLab Corporation, Northampton, MA, USA) was used to fit the electropherograms.
